# Outcome of Conversion Surgery After Induction Therapy for Esophageal Cancer with Synchronous Para-Aortic Lymph Node Metastasis: A Multi-institutional Retrospective Study

**DOI:** 10.1245/s10434-025-18042-w

**Published:** 2025-08-20

**Authors:** Koji Tanaka, Keijiro Sugimura, Norihiro Matsuura, Osamu Shiraishi, Kota Momose, Kotaro Yamashita, Takahito Sugase, Takashi Kanemura, Tomoki Makino, Atsushi Takeno, Ryohei Kawabata, Masaaki Motoori, Yutaka Kimura, Makoto Yamasaki, Hiroshi Miyata, Kazumasa Fujitani, Takushi Yasuda, Masahiko Yano, Hidetoshi Eguchi, Yuichiro Doki

**Affiliations:** 1 Department of Gastroenterological Surgery, Graduate School of Medicine, Osaka University, Suita, Osaka Japan; 2 Department of Surgery, Kansai Rosai Hospital, Hyogo, Japan; 3 Department of Surgery, Osaka International Cancer Institute, Osaka, Japan; 4 Department of Surgery, Kindai University Faculty of Medicine, Osaka Sayama, Osaka Japan; 5 Department of Surgery, National Hospital Organization, Osaka National Hospital, Osaka, Japan; 6 Department of Surgery, Sakai City Medical Center, Osaka, Japan; 7 Department of Surgery, Osaka General Medical Center, Osaka, Japan; 8 Department of Gastroenterological Surgery, Kindai University Nara Hospital, Nara, Japan; 9 Department of Surgery, Kansai Medical University, Hirakata, Osaka Japan; 10 Kyowakai Hospital, Osaka, Japan

**Keywords:** Esophageal cancer, Conversion surgery, Para-aortic lymph node

## Abstract

**Background:**

Systemic chemotherapy is the standard treatment for esophageal cancer with synchronous distant metastasis including para-aortic lymph node (PALN) metastasis. The significance of conversion surgery for esophageal cancer with synchronous PALN metastasis remains controversial.

**Objective:**

The current study aimed to investigate the clinical outcome of conversion surgery for esophageal cancer with synchronous PALN metastasis after induction therapy.

**Methods:**

This multi-institutional retrospective study included 48 patients with esophageal cancer who exhibited synchronous PALN metastasis and who received induction chemotherapy or chemoradiotherapy followed by conversion surgery between 2005 and 2022. The short- and long-term treatment outcomes were examined.

**Results:**

Among the 48 patients, 45 and 3 received chemotherapy and chemoradiotherapy, respectively, as the initial treatment. Moreover, all patients underwent subtotal esophagectomy. The incidence rate of postoperative complications was 48% and the in-hospital mortality rate was 2%. The 3- and 5-year overall survival rates of all patients were 36.1% and 25.2%, respectively. The overall survival rates of patients with pN2-3 and final PALN status (fM1) were significantly lower than that of patients with pN0-1 (*p* = 0.0025) and fM0 (*p* = 0.0043). The multivariate analysis showed that pathological nodal status (hazard ratio 2.44, *p* = 0.0488) and fM status (hazard ratio 2.53, *p* = 0.0246) were independent prognostic factors.

**Conclusions:**

Conversion surgery for esophageal cancer with synchronous PALN metastasis is feasible and promising. In addition, conversion surgery for patients with controlled nodal status including PALN metastasis is important for long-term prognosis.

**Supplementary Information:**

The online version contains supplementary material available at 10.1245/s10434-025-18042-w.

Esophageal cancer with para-aortic lymph node (PALN) metastasis is classified as stage IVb disease, which, historically, has a dismal 5-year survival rate of approximately 10%.^1^ Esophageal cancer with PALN metastasis is traditionally considered a systemic disease with poor prognosis. In fact, the presence of PALN metastasis is often associated with a higher number of metastatic lymph nodes and worse survival outcomes compared with the absence of PALN involvement.^1^ Furthermore, patients with pathological abdominal PALN metastasis are more likely to exhibit hematogenous recurrence than those without. This finding indicated that abdominal PALN metastasis can lead to systemic disease. Systemic chemotherapy or immunotherapy is the standard treatment for advanced-stage esophageal cancer with synchronous distant metastasis, including PALN metastasis; however, recent advancements in multimodal treatment strategies have led to the exploration of more aggressive approaches, including conversion surgery, in some patients.

Recent studies have challenged the notion that all patients with PALN metastasis from esophageal cancer are not suitable candidates for surgical intervention. In various gastrointestinal malignancies, the concept of conversion surgery, which involves systemic therapy followed by surgical resection in initially unresectable cases, has gained attention. However, the majority of studies are case reports,^2–4^ and there are only a few researches on the outcome of conversion surgery in patients with PALN metastasis in a somewhat large cohort.^5–8^ Although preliminary, these results indicate that a subset of patients with PALN metastasis may benefit from this aggressive approach. The rationale behind conversion surgery lies in the potential of systemic therapy to downstage the disease, thereby allowing for subsequent surgical resection with curative intent. This strategy aims to address both local and systemic disease control. In particular, subtotal esophagectomy is an extremely invasive surgery, and data on the safety and therapeutic efficacy of conversion surgery are currently insufficient.

As for the systemic control of esophageal cancer, the combination of two drugs, cisplatin and 5-fluorouracil, has long been the standard chemotherapeutic regimen for synchronous distant metastasis from esophageal cancer. Triplet chemotherapy such as cisplatin and fluorouracil plus adriamycin (ACF) and cisplatin and fluorouracil plus docetaxel (DCF), which is a more potent neoadjuvant chemotherapy, has been developed for patients with resectable advanced-stage esophageal cancer.^9–13^ In practice, these triplet chemotherapeutic regimens are occasionally used in patients with PALN metastasis from stage 4 esophageal cancer; however, there is also no consensus on their outcomes. In recent years, the efficacy of immune checkpoint inhibitors (ICIs) against esophageal cancer has also been observed. Based on the results of two large, prospective, randomized trials, cisplatin and 5-fluorouracil plus ICIs or dual ICIs comprising nivolumab and ipilimumab were associated with a better survival than the combination of cisplatin and 5-fluorouracil for the treatment of unresectable or recurrent esophageal cancer.^14^ Based on the abovementioned data, at present, cisplatin and 5-fluorouracil plus ICIs or dual checkpoint inhibitors with nivolumab and ipilimumab are considered the standard treatments for esophageal cancer with synchronous distant metastasis.

However, the selection of appropriate candidates for conversion surgery remains challenging and factors such as response to induction therapy, extent of lymph node involvement, and chemotherapeutic regimen must be cautiously considered. As the landscape of esophageal cancer treatment evolves, with advancements in systemic therapies and surgical techniques, there is a growing need to reassess the role of surgical intervention in patients with PALN metastasis.

Thus, the current study assessed the outcomes of conversion surgery for esophageal cancer with synchronous PALN metastasis after induction systemic therapy in a large patient cohort. Moreover, the clinical impact of conversion surgery for esophageal cancer with synchronous PALN metastasis was investigated.

## Methods

### Patients

Data were collected from the medical records of 3443 consecutive patients with esophageal cancer who underwent esophagectomy between 2005 and 2021 at five institutions. Among all patients, 53 presented with synchronous PALN metastases, of whom one with synchronous PALN who had liver metastasis, three who underwent esophagectomy without preoperative treatment, and one whose histology was not squamous cell carcinoma or adenocarcinoma were excluded from the analysis. The current study included the remaining 48 patients who underwent esophagectomy after induction treatment. All patients were pathologically diagnosed with squamous cell carcinoma or adenocarcinoma based on the assessment using pretreatment biopsy samples. This study was approved by the appropriate Institutional Review Boards of Osaka University Hospital (approval number 16305-3) and was conducted in accordance with the Declaration of Helsinki.

### Assessment of Clinical Staging

All patients were staged before and after surgery according to the Eighth Edition of the Union for International Cancer Control (UICC) staging system.^15^ Clinical staging before preoperative treatment was based on the endoscopy, neck, chest, and abdominal computed tomography (CT) scan. A positron emission tomography (PET) scan was performed in some patients at the discretion of each institution. Patients with lymph nodes that were spherical and had a maximum transverse diameter of >1.0 cm on CT scan, or those who presented with focal major ^18^F-2-deoxy-d-glucose (FDG) uptake, compared with normal mediastinal activity, on PET scan were considered as metastasis-positive. Patients with lymph nodes that were visible but smaller than 1.0 cm on the long axis on CT scan were regarded as metastasis-positive only if prominent FDG uptake was detected. CT and PET/CT findings were interpreted via a double-check process by surgeons/oncologists and a radiologists.

### Definition of Para-Aortic Lymph Node (PALN)

Herein, abdominal PALNs (#16, as defined by the Japanese Classification of Esophageal Cancer^19,20^) were considered as PALNs, including lateral and internal PALNs. Thoracic para-aortic nodes (#112AoP, as defined by the Japanese Classification of Esophageal Cancer^19,20^) were not classified as PALN.

### Induction Treatment

All patients initially had esophageal cancer with PALN metastasis. They were then treated with initial chemotherapy or chemoradiotherapy. The treatment regimens were classified into three groups: (1) triplet chemotherapy regimen;^9–13^ (2) doublet chemotherapy regimen;^16^ and (3) radiation-containing regimens.^17,18^

### Surgical Procedure

The standard surgical procedure for thoracic esophageal cancer comprised subtotal esophagectomy with mediastinal lymphadenectomy, which was performed according to the Japanese Classification of Esophageal Cancer,^19,20^ via a right thoracotomy or a robot-assisted thoracoscopy.^21–25^ A transhiatal lower esophagectomy was conducted on several patients with lower-thoracic esophageal cancer. After the thoracic procedure, patients were repositioned in the supine position and abdominal lymphadenectomy with or without PALN resection1 and gastric conduit reconstruction were performed. Jejunal or colonic graft reconstruction was conducted on patients with a history of gastrectomy.^26–28^ PALN resection generally removed PALNs between the upper margin of the origin of the celiac artery and the lower border of the left renal vein, and PALNs between the lower border of the left renal vein and upper border of the origin of the inferior mesenteric artery, according to the Japanese Classification of Esophageal Cancer.^19,20^ The type of surgical procedure (esophagectomy or reconstruction, and extent of PALN resection) was determined by the responsible surgeon. Basically, to balance operative morbidity with oncologic benefit, anatomical resection was performed for PALN that persisted on imaging. Meanwhile, PALNs with complete or remarkable radiographic response were sampled for pathological confirmation (electronic supplementary material Fig. 1). The Clavien–Dindo classification system was used to assess complications. Grade 3 was defined as complications that need surgical, endoscopic, or radiologic intervention;^29^ grade 4 was defined as a life-threatening complication requiring intensive care unit management; and grade 5 was defined as a complication causing death. Patients with grade 2 or higher complications were considered to have complications.

**Fig. 1:**
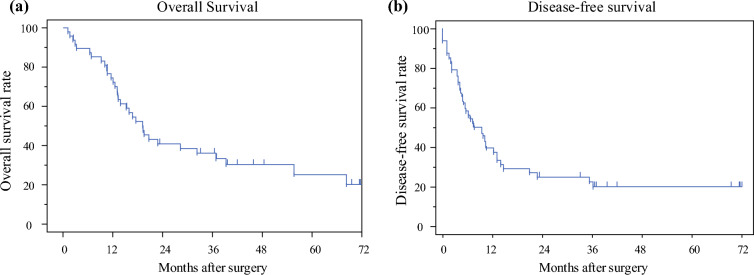
**a** Kaplan–Meier estimates of OS. The 3- and 5-year OS rates of all patients were 36.1% and 25.2%, respectively. **b** Kaplan–Meier estimates of DFS. The 3- and 5-year DFS rates were 20.0% and 20.0%, respectively. *OS* overall survival, *DFS* disease-free survival

### Evaluation of Clinical and Pathological Response

Clinical response was evaluated based on CT scan and endoscopy images according to the World Health Organization response criteria for measurable disease and the Japanese Classification of Esophageal Cancer criteria.^19,20^ Clinical response was classified into four categories: complete response, partial response, stable disease, or progressive disease. Patients who achieved complete or partial response were categorized as responders. Meanwhile, patients who achieved stable disease or progressive disease were categorized as non-responders. Pathological stage was determined according to the Eighth Edition of the UICC classification system, and pathological response was categorized according to the Japanese Society for Esophageal Diseases criteria. Viable residual tumor cells within the whole tumor were graded as follows: grade 3, no viable residual tumor cells; grade 2, few residual tumor cells; grade 1b, fewer than two-thirds of residual tumor cells; grade 1a, greater than two-thirds of residual tumor cells; and grade 0, no significant response to preoperative treatment.

### Follow-Up Evaluation

All patients were assessed at the outpatient clinic at intervals of 3–4 months within the first 2 years and every 6 months for the following 3 years. CT scan images and tumor marker levels were evaluated every 3–4 months within the first 2 years and every 6 months for the following 3 years. Annual upper gastrointestinal endoscopy was performed to screen for recurrence at the anastomotic site and in the gastric conduit. If the CT scan results indicated recurrence, further investigations were performed using more selective methods (e.g., PET, bone scintigraphy, and magnetic resonance imaging).

### Statistical Analysis

Quantitative results were expressed as the mean ± standard deviation. Between-group differences were examined using Student’s *t*-test with Yates’ correction, Chi-square test, Fisher’s exact probability test, or Mann–Whitney U test, as applicable. The overall survival (OS) rate was calculated from the date of surgery to the date of death or last known date of follow-up, while the disease-free survival (DFS) rate was calculated from the date of surgery to the date of recurrence or death or last known date of follow-up. Survival curves were plotted using the Kaplan–Meier method, and differences between survival curves were compared using the log-rank test. To determine the prognostic factors, a multivariate analysis was performed using the Cox proportional hazards model. Prognostic variables were introduced into the model if the univariate analysis demonstrated a significance level of *p* < 0.2. *P*-values <0.05 indicated statistically significant differences. Statistical analysis was performed using JMP version 17.0 (SAS Institute, Cary, NC, USA).

## Results

### Patients

Table 1 shows the characteristics of all patients who were included in this study. In total, 42 (87%) patients had clinical T3 factors or higher and 35 (63%) presented with cN2 or cN3 factors. Of 48 patients who underwent salvage surgery for PALN metastases, 18 (37.5%) presented with a solitary metastatic node and 30 (62.5%) presented with multiple metastatic nodes, with the maximum number of involved nodes in a single patient being six. All patients received induction treatment. The number of regimens received before conversion surgery was 1 (*n* = 41, 85%) and 2 (*n* = 7, 15%). Nine (19%) patients had received radiotherapy. The initial treatment comprised triple chemotherapy, such as ACF or DCF, in 41 (85%) patients; dual chemotherapy, including FP, in 4 (8%) patients; and chemoradiotherapy in 3 (6%) patients. In total, nine patients received chemoradiotherapy (CRT) before surgery—three as initial induction therapy (including PALN fields in three cases) and six as subsequent salvage therapy for locoregional control of advanced-stage esophageal cancer (in whom PALN stations were not included in the radiation field). The median time from treatment initiation to conversion surgery was 77 days (range 31–872). The clinical therapeutic effects at the time of conversion surgery were complete response (*n* = 1, 2%), partial response (*n* = 36, 75%), stable disease (*n* = 5, 10%), and progressive disease (*n* = 2, 4%).

**Table 1:** Participant characteristics [*N* = 48]

Characteristics		
Age, years [mean (range)]		64 (43–76)
Sex	Male	38 (79)
	Female	10 (21)
Tumor location	Upper	1 (2)
	Middle	19 (40)
	Lower	28 (58)
Histology	Squamous cell carcinoma	43 (90)
	Adenocarcinoma	5 (10)
Clinical T stage	cT2	6 (13)
	cT3	29 (60)
	cT4a	2 (4)
	cT4b	11 (23)
Clinical N stage	cN0	0 (0)
	cN1	13 (27)
	cN2	23 (48)
	cN3	12 (25)
Clinical M stage	cM1	48 (100)
No. of PALN metastases	Single	26 (54)
	Multiple	22 (46)
Clinical stage	IV	48 (100)
No. of regimens in the induction treatment	0	0 (0)
	1	41(85)
	2	7 (15)
Radiation therapy	Yes	9 (19)
	No	39 (81)
Initial treatment	Chemotherapy	45 (93)
	Triplet regimen	41 (85)
	Doublet regimen	4 (8)
	Chemoradiotherapy	3 (6)
Clinical response to induction therapy	Complete response	1 (2)
	Partial response	36 (75)
	Stable disease	5 (10)
	Progressive disease	2 (4)
	Not available	4 (8)

Data are expressed as *n* (%) unless otherwise specified

*PALN* para-aortic lymph node

### Surgical and Pathological Findings

Table 2 depicts the operative and pathological findings. For conversion surgery, 30 (62%) patients underwent subtotal esophagectomy via thoracotomy, 16 (34%) underwent thoracoscopic esophagectomy, and 2 (4%) underwent robot-assisted esophagectomy. In total, 46 (96%) patients underwent one-stage resection and reconstruction and 2 (4%) patients underwent two-stage surgery. Systematic anatomical dissection was performed on 23 patients and sampling was performed on 20 patients. PALN resection was not conducted on five patients with complete clinical response. Systemic dissection or sampling of PALN metastatic lesions was performed on 43 (90%) patients. Pathologically, R0 resection was conducted on 45 (94%) patients. In total, 30 (63%) patients presented with ypN2 stage or higher, and 21 (44%) patients with ypM1 were pathologically found to exhibit PALN metastasis. In terms of the final PALN status (fM1), which was defined according to the Japanese Society for Esophageal Diseases criteria,^19,20^ 27 patients were PALN-negative.

**Table 2:** Surgical procedures and pathological examination results [*N* = 48]

Variables		
Surgical procedure	Subtotal esophagectomy	48 (100)
	Thoracotomy	30 (62)
	Thoracoscopy	16 (34)
	Robot-assisted	2 (4)
Type of surgery	Primary	46 (96)
	Two-staged	2 (4)
Lymphadenectomy	Two-field	21 (44)
	Three-field	27 (56)
Organ reconstruction	Gastric conduit	45 (94)
	Pedicled jejunum	3 (6)
	Pedicled colon	0 (0)
Reconstruction route	Retrosternal	24 (50)
	Posterior mediastinal	14 (29)
	Percutaneous	10 (21)
Resection of PALN metastatic lesions	Yes	43 (90)
	No	5 (10)
Surgical duration, min [mean ± SD]	597 ± 143	
Blood loss volume, mL [mean ± SD]	863 ± 725	
Pathological T stage	ypT0	9 (19)
	ypT1	7 (15)
	ypT2	10 (21)
	ypT3	20 (42)
	ypT4	2 (4)
Pathological N stage	ypN0	11 (23)
	ypN1	7 (15)
	ypN2	15 (32)
	ypN3	15 (31)
Pathological M stage	ypM0	22 (46)
	ypM1	21 (44)
	Not resected	5 (10)
Pathological stage	ypStage 0	5 (10)
	ypStage I	4 (8)
	ypStage II	1 (2)
	ypStage III	13 (27)
	ypStage IV	25 (52)
Histological grade	0	1 (2)
	1a	16 (33)
	1b	7 (15)
	2	14 (29)
	3	10 (21)
Operative curability	R0	45 (94)
	R2	3 (6)
Final PALN status	PALN-negative	27 (56)
	PALN-positive	21 (44)

Data are expressed as *n* (%) unless otherwise specified

*PALN* para-aortic lymph node, *SD* standard deviation

### Surgical Outcomes and Postoperative treatment

Table 3 shows the short-term outcomes of surgery. In total, 23 (48%) patients developed postoperative complications. In particular, eight (20%) patients presented with postoperative pulmonary complications, three (6%) presented with anastomotic leakage, and 3 (6%) presented with recurrent laryngeal nerve palsy. One (2%) patient underwent reoperation for chylothorax, and there was one (1%) case of postoperative in-hospital mortality due to acute respiratory distress syndrome. Adjuvant therapy was administered to 19 (40%) patients (fluoropyrimidine monotherapy, *n* = 5; taxane monotherapy, *n* = 6; doublet such as 5-fluorouracil and cisplatin, *n* = 2; CRT, *n* = 2; and ICI-based therapy, *n* = 4). These regimens were individualized based on pathologic risk and patient condition.

**Table 3:** Morbidity and mortality after salvage esophagectomy [*N* = 48]

Complications	
Any	23 (48)
Pulmonary	8 (20)
Anastomotic leakage	3 (6)
Recurrent laryngeal nerve palsy	3 (6)
Bleeding	0 (0)
Cardiovascular	1 (2)
Chylothorax	5 (10)
Abdominal lymphatic fistula	1 (2)
Empyema	0 (0)
Enteritis	1 (2)
Others	1 (2)
Re-operation	1 (2)
In-hospital mortality	1 (2)

Data are expressed as *n* (%)

### Survival Analysis

**Fig. 2:**
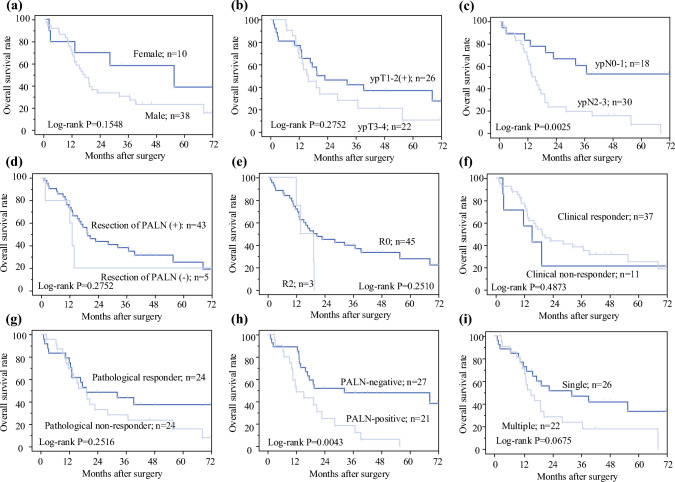
Kaplan–Meier estimates of overall survival based on **a** sex, **b** pathological ypT stage, **c** pathological ypN stage, **d** history of resection of PALN metastatic lesions, **e** history of R0 resection, **f** clinical response to induction therapy, **g** pathological response to induction therapy for the primary tumor, **h** final PALN status, and **i** number of PALN metastases. *PALN* para-aortic lymph node

The 3- and 5-year OS rates of all patients who were included in the current study were 36.1% and 25.2%, respectively, and the median OS time was 19 months (Fig. 1a). The 3- and 5-year DFS rates were 20.0% and 20.0%, respectively, and the median DFS time was 8 months (Fig. 1b). There was no significant association between survival and sex, clinical TNM factors, pathological T factor, history of distant metastatic lesion resection and R0 resection, clinical therapeutic effects on the primary tumor, and histological effect on the primary tumor (Figs. 2a, b, d, e, f, and g). By contrast, in pathological N stage, the prognosis of the ypN0-1 group was significantly better than that of the ypN2-3 group (3-year OS: 60.6% vs. 23.6%, *p* = 0.0025) (Fig. 2b). Regarding the final PALN status, the prognosis of the PALN(−) group was significantly better than that of the PALN(+) group (3-year OS: 47.9% vs. 24.8%, *p* = 0.0043) (Fig. 2h). Based on the number of PALN metastatic lesions, the prognosis of the single clinical PALN metastasis (cPALN) group was better than that of the multiple cPALN group; however, the results did not significantly differ (*p* = 0.0675) (Fig. 2i). In total, 27 patients had disease confined to stations 16a, and 21 had lesions extending into 16b1/b2. The median OS of the two subgroups (16a vs. 16b1/b2) was comparable (*p* = 0.2951). The multivariate analysis showed that pathological nodal stage (≥ypN2) [hazard ratio (HR) 2.69, *p* = 0.0256] and final PALN status (PALN-positive) [HR 2.34, *p* = 0.0320] were independent prognostic factors (Table 4).

**Table 4:** Factors associated with overall survival in univariate and multivariate analyses

Variables	Univariate analysis			Multivariate analysis		
	HR	95% CI	*p*-Value	HR	95% CI	*p*-Value
Age (≥70 years)	0.95	0.41–2.22	0.9134			
Male sex	2.01	0.77–5.24	0.1548	2.26	0.82–6.22	0.1136
Location (lower)	1.48	0.73–3.03	0.2798			
Clinical T stage (≥cT4)	1.57	0.73–3.40	0.2500			
Clinical N stage (≥cN2)	0.80	0.38–1.67	0.5505			
No. of PALN metastases (multiple)	1.92	0.95–3.85	0.0675	1.18	0.56–2.46	0.6674
Preoperative treatment interval (≥90 days)	1.33	0.66–2.69	0.4257			
Clinical response to preoperative treatment (non-responder)	1.41	0.54–3.67	0.4873			
Resection of PALN (non-resection)	1.52	0.53–4.39	0.4353			
Pathological T stage (≥pT3)	1.47	0.74–2.93	0.2752			
Pathological N stage (≥pN2)	3.51	1.55–7.95	0.0025	2.69	1.13–6.40	0.0256
Final PALN status (PALN-positive)	2.82	1.38–5.73	0.0043	2.34	1.08–5.08	0.0320
Pathological response to preoperative treatment (non-responder)	1.50	0.75–3.00	0.2516			
Resection margin (R2)	2.03	0.61–6.82	0.2510			
Postoperative treatment (no)	1.06	0.52–2.14	0.8734			

Cox proportional hazards models were used in the univariate and multivariate analyses of overall survival in 66 patients who underwent salvage esophagectomy

*CI* confidence interval, *HR* hazard ratio, *PALN* para-aortic lymph node

### Recurrence Pattern

The recurrence pattern in 45 patients who achieved R0 resection was investigated. Among these 45 patients, 35 (77.8%) had recurrence. As for lymph node recurrence, PALNs were observed in 18 (38%) cases, regional lymph nodes were observed in 10 (21%) cases, and other non-regional lymph nodes were observed in 8 (17%) cases. Furthermore, 10 (20%) patients presented with liver metastasis, 4 (8.5%) presented with peritoneal dissemination, and 2 (4.3%) presented with lung metastasis.

## Discussion

This multi-institutional retrospective analysis showed that conversion surgery after induction therapy was a paradigm-shifting approach for patients with esophageal cancer who exhibited synchronous PALN metastasis, with a 5-year OS rate of 25.2% in cautiously selected candidates. The survival outcomes can challenge the traditional perceptions of PALN metastasis as universally terminal while revealing critical prognostic determinants that may guide therapeutic decision making.

As for the feasibility and safety of conversion surgery, the postoperative complication rate in our study was 48% and the in-hospital mortality rate was low at 2%. Notably, systemic PALN dissection/sampling was performed in 90% of cases, thereby reflecting the technical complexity of these procedures. An R0 resection rate of 94% underscores the importance of meticulous surgical planning, particularly in addressing residual PALN metastases after induction therapy. These results are comparable with those reported in other studies on conversion surgery for advanced-stage esophageal cancer. For example, Igaue et al. reported similar short-term safety outcomes between patients with and without resectable M1 lymph node metastatic lesions who underwent esophagectomy.^6^ Similarly, according to Tsuji et al., who performed a multi-institutional study of conversion therapy for esophageal squamous cell carcinoma with distant metastasis, the postoperative complication rate was 47%.^30^ Based on these findings, conversion surgery can be performed safely in cautiously selected patients, despite the disease being in the advanced stage. Our study showed that conversion surgery for esophageal cancer with synchronous PALN metastasis was feasible; however, the limitations of the current imaging techniques in accurately assessing PALN status should be considered. The sampling of PALNs during surgery may provide valuable information for guiding postoperative adjuvant therapy decision making. Nevertheless, patients with pathologically confirmed PALN metastases have a poor prognosis. Considering the limited survival benefit and the risk for increased surgical morbidity, routine extensive PALN dissection or sampling may not be required for all patients undergoing conversion surgery. The decision to perform PALN dissection or sampling should be carefully balanced against the potential risks and benefits of each individual patient.

Our multivariate analysis revealed that pathological nodal status (≥ypN2) and final PALN status (PALN-positive) were independent prognostic factors. Notably, pathological response in primary tumors was not correlated with survival (*p* = 0.435), indicating systemic biology rather than local response dictating prognosis. This result is in accordance with the findings of other studies. Shigeno et al. performed a study on conversion surgery for esophageal squamous cell carcinoma with solitary abdominal PALN metastasis and reported that pathological responders had a significantly longer OS than non-responders.^5^ Similarly, Igaue et al. found that ypN status was the only independent prognostic factor in their analysis of cases involving the resection of M1 lymph node metastatic lesions.^6^ Our results confirm that both overall lymph node involvement and residual PALN metastases after systemic therapy are associated with decreased survival. Consequently, conversion surgery should be reserved for patients whose non-para-aortic nodal disease is controlled and whose PALN lesions have resolved on imaging. In these patients, a pathologically negative PALN may indicate that anatomical para-aortic dissection is unnecessary. Conversely, when PALNs remain pathologically positive, resection primarily serves to establish the presence of residual metastasis and inform prognosis, rather than to confer a direct survival benefit by local control of PALN. Although complete PALN clearance is a prerequisite for local disease control, 60% of recurrences occur outside the para-aortic region. This pattern underscores the potential role for more intensive systemic approaches—particularly ICIs—to address micrometastatic disease beyond the surgical field.

The results of our study and others in the literature emphasize the importance of patient selection for conversion surgery. Patients who have a good response to induction therapy, particularly in terms of nodal status, are the best candidates for this approach. The choice of induction therapy regimen may also play a role in outcomes. Our study primarily used triplet chemotherapy regimens. Nevertheless, recent advancements in systemic therapies, including the use of ICIs, may further improve response rates and survival outcomes. Moreover, the integration of novel biomarkers and advanced imaging techniques may help refine patient selection and predict response to induction therapy. However, our analysis also revealed that patients with persistent pathological involvement of PALNs (ypM1) at the time of surgery had poor long-term survival outcomes. This finding underscores the limitations of conversion surgery in eradicating systemic diseases in patients with inadequate response to induction therapy. Considering the dismal prognosis associated with persistent pathological PALN involvement, we believe that conversion surgery should not be routinely offered to patients with PET-positive disease, as these individuals are unlikely to experience a significant survival benefit from surgical resection. Future research should focus on developing more effective induction regimens and refining patient selection criteria to identify individuals who are most likely to benefit from conversion surgery. Circulating tumor DNA (ctDNA) has emerged as a promising tool for monitoring several cancers, including esophageal cancer. ctDNA has a high diagnostic performance in advanced-stage esophageal cancer, and monitoring dynamic changes in ctDNA has been found to be beneficial for evaluating therapeutic efficacy and predicting early recurrence in esophageal cancer.^31–34^ This approach enables clinicians to assess treatment response more accurately and adjust therapies as needed. The potential role of ctDNA in monitoring treatment response and guiding decision making for conversion surgery must be further investigated.

Our study had several limitations. In particular, a significant constraint was the inherent selection bias attributed to the retrospective nature of the research and the inclusion of patients who underwent resection only. Hence, it is important to emphasize that our cohort represented a highly selected group of patients who had favorable responses to induction therapy and were considered suitable for surgery. Although encouraging, the survival outcomes reported in this research should be interpreted in the context of this selection bias. Even in this cautiously curated group of patients, the achievement of long-term survival remains challenging, thereby underscoring the aggressive nature of esophageal cancer with initial PALN metastases. This retrospective multi-institutional study is subject to selection bias due to variable patient selection, induction regimens, and surgical indications across centers. Each center’s multidisciplinary tumor board determined surgical candidacy based on clinical response, performance status, and resectability. Variability across centers reflects real-world practice and introduces selection bias. Heterogeneity in timing to surgery (range 31–872 days) and PALN management should be considered. Absence of a contemporaneous non-conversion cohort for direct comparison is also a key limitation of the present study. Our institutional database does not systematically record detailed information of patients deemed unsuitable for surgery, and selection criteria for non-surgical management (e.g., comorbidity profile, unresectable disease extent) differ in ways that cannot be retrospectively harmonized. Prospective, multicenter registries or randomized studies are needed to definitively quantify the survival benefit attributable to conversion surgery. In addition, the relatively small sample size limited the generalizability of our findings.

Indeed, CT and PET/CT, although integral to preoperative staging, have inherent limitations concerning sensitivity and specificity for lymph node metastasis. CT is dependent on size and morphological criteria, which are not reliable when used to distinguish reactive enlargement from true metastatic involvement. Conversely, micrometastatic disease in subcentimeter nodes may be missed. PET/CT uses FDG uptake as a surrogate for malignancy; however, it may produce false-positive signals in inflamed or fibrotic nodes, particularly after chemoradiation, and false-negative signals for small or low metabolic activity metastases. Furthermore, neoadjuvant systemic therapy can induce complete pathological response in previously involved lymph nodes. Nodes that appeared positive on pretreatment imaging may only show necrosis and fibrosis at surgery, yielding a pathological negative result despite initial metastatic involvement. Therefore, in our cohort, some nodes classified as pathologically negative likely represent either false-positive imaging or true metastases eradicated by preoperative therapy, rather than nodes that were never involved. This distinction underscores the need for cautious interpretation of imaging-pathology concordance and suggests that pathological negativity after neoadjuvant treatment does not necessarily imply the absence of previous metastatic disease.

The study results are from an era when ICI was not used. Currently, based on the results of the Keynote-590^35^ and Checkmate-648 trials,^14^ ICI-based systemic therapy is the standard initial treatment for esophageal cancer with synchronous distant metastasis. Therefore, in the future, when ICI is used as the initial treatment, the position of conversion surgery may change. Nevertheless, future prospective studies with larger cohorts and standardized treatment protocols should be performed to further validate the role of conversion surgery in this patient population.

## Conclusion

Conversion surgery after induction therapy for esophageal cancer with synchronous PALN metastasis is feasible and can lead to favorable long-term outcomes in some patients. Cautious patient selection based on response to induction therapy and pathological nodal status is essential for optimizing results. However, patients with persistent pathological PALN involvement at the time of surgery had a poor prognosis, with limited long-term survival. Therefore, conversion surgery is not recommended for patients with persistent PALN-positive status such as PET-positive disease before surgery. This is because these individuals are unlikely to experience a significant survival benefit from surgical resection. As systemic therapies continue to evolve, including the integration of immunotherapy, the landscape of treatment for advanced-stage esophageal cancer is likely to change, potentially expanding the role of conversion surgery in the future.

## Supplementary Information

Below is the link to the electronic supplementary material.

Supplementary file1 (JPG 988 kb)

## References

[1] Tanaka K, Yano M, Motoori M, et al. The significance of abdominal para-aortic lymph node metastasis in patients with lower thoracic esophageal cancer. *Dis Esophagus*. 2012;25(2):146–52. 21762280 10.1111/j.1442-2050.2011.01222.x

[2] Kawata A, Miyamoto Y, Fukubayashi K, et al. Conversion surgery after encorafenib plus cetuximab for chemorefractory BRAF V600E-mutated colorectal cancer with para-aortic lymph node metastases. *In Vivo*. 2023;37(4):1797–801. 37369457 10.21873/invivo.13269 PMC10347918

[3] Serizawa A, Taniguchi K, Yamada T, et al. Successful conversion surgery for unresectable gastric cancer with giant para-aortic lymph node metastasis after downsizing chemotherapy with S-1 and oxaliplatin: a case report. *Surg Case Rep*. 2018;4(1):88. 30088107 10.1186/s40792-018-0494-4 PMC6081489

[4] Ushimaru Y, Nishikawa K, Yasuhara Y, et al. Successful laparoscopic conversion surgery for gastric cancer with para-aortic lymph node metastasis after third-line chemotherapy: a case report. *Int Cancer Conf J*. 2022;11(1):50–6. 34660169 10.1007/s13691-021-00516-9 PMC8511852

[5] Shigeno T, Kajiyama D, Sato K, et al. Efficiency of conversion surgery for esophageal squamous cell carcinoma with solitary abdominal para-aortic lymph node metastasis. *Surg Today*. 2024;54(12):1490–7. 38802718 10.1007/s00595-024-02872-4

[6] Igaue S, Nozaki R, Utsunomiya D, et al. Significance of surgery for resectable M1 lymph node metastases without organ metastasis in esophageal carcinoma in the era of neoadjuvant treatment. *Ann Surg Oncol*. 2024;31(3):1525–35. 37996638 10.1245/s10434-023-14562-5

[7] Marrelli D, Piccioni SA, Carbone L, et al. Posterior and para-aortic (D2plus) lymphadenectomy after neoadjuvant/conversion therapy for locally advanced/oligometastatic gastric cancer. *Cancers*. 2024;16(7):1376. 38611054 10.3390/cancers16071376 PMC11010857

[8] Yoshida K, Yasufuku I, Terashima M, et al. International retrospective cohort study of conversion therapy for Stage IV Gastric Cancer 1 (CONVO-GC-1). *Ann Gastroenterol Surg*. 2022;6(2):227–40. 35261948 10.1002/ags3.12515 PMC8889854

[9] Yamasaki M, Yasuda T, Yano M, et al. Multicenter randomized phase II study of cisplatin and fluorouracil plus docetaxel (DCF) compared with cisplatin and fluorouracil plus Adriamycin (ACF) as preoperative chemotherapy for resectable esophageal squamous cell carcinoma (OGSG1003). *Ann Oncol*. 2017;28(1):116–20. 27687307 10.1093/annonc/mdw439

[10] Yamasaki M, Miyata H, Tanaka K, et al. Multicenter phase I/II study of docetaxel, cisplatin and fluorouracil combination chemotherapy in patients with advanced or recurrent squamous cell carcinoma of the esophagus. *Oncology*. 2011;80(5–6):307–13. 21778771 10.1159/000329806

[11] Kato K, Machida R, Ito Y, et al. Doublet chemotherapy, triplet chemotherapy, or doublet chemotherapy combined with radiotherapy as neoadjuvant treatment for locally advanced oesophageal cancer (JCOG1109 NExT): a randomised, controlled, open-label, phase 3 trial. *Lancet*. 2024;404(10447):55–66. 38876133 10.1016/S0140-6736(24)00745-1

[12] Yamasaki M, Yasuda T, Yano M, et al. Multicenter randomized phase II study of cisplatin and fluorouracil plus docetaxel (DCF) compared with cisplatin and fluorouracil plus Adriamycin (ACF) as preoperative chemotherapy for resectable esophageal squamous cell carcinoma (OGSG1003). *Ann Oncol*. 2017;28(1):116–20. 27687307 10.1093/annonc/mdw439

[13] Yamashita K, Katada N, Moriya H, et al. Neoadjuvant chemotherapy of triplet regimens of docetaxel/cisplatin/5-FU (DCF NAC) may improve patient prognosis of cStage II/III esophageal squamous cell carcinoma-propensity score analysis. *Gen Thorac Cardiovasc Surg*. 2016;64(4):209–15. 26868531 10.1007/s11748-016-0626-3

[14] Doki Y, Ajani JA, Kato K, et al. Nivolumab combination therapy in advanced esophageal squamous-cell carcinoma. *N Engl J Med*. 2022;386(5):449–62. 35108470 10.1056/NEJMoa2111380

[15] Brierley JD, Gospodarowicz MK, Wittekind C. TNM classification of malignant tumours. Amsterdam: Wiley; 2017.

[16] Shinoda M, Ando N, Kato K, et al. Randomized study of low-dose versus standard-dose chemoradiotherapy for unresectable esophageal squamous cell carcinoma (JCOG0303). *Cancer Sci*. 2015;106(4):407–12. 25640628 10.1111/cas.12622 PMC4409884

[17] Ohtsu A, Boku N, Muro K, et al. Definitive chemoradiotherapy for T4 and/or M1 lymph node squamous cell carcinoma of the esophagus. *J Clin Oncol*. 1999;17(9):2915–21. 10561371 10.1200/JCO.1999.17.9.2915

[18] Higuchi K, Komori S, Tanabe S, et al. Definitive chemoradiation therapy with docetaxel, cisplatin, and 5-fluorouracil (DCF-R) in advanced esophageal cancer: a phase 2 trial (KDOG 0501–P2). *Int J Radiat Oncol Biol Phys*. 2014;89(4):872–9. 24867539 10.1016/j.ijrobp.2014.03.030

[19] Mine S, Tanaka K, Kawachi H, et al. Japanese classification of esophageal cancer, 12th Edition: Part I. *Esophagus*. 2024;21(3):179–215. 38568243 10.1007/s10388-024-01054-y PMC11199297

[20] Doki Y, Tanaka K, Kawachi H, et al. Japanese classification of esophageal cancer, 12th Edition: Part II. *Esophagus*. 2024;21(3):216–69. 38512393 10.1007/s10388-024-01048-w PMC11199314

[21] Cuesta MA, van der Wielen N, Straatman J, van der Peet DL. Video-assisted thoracoscopic esophagectomy: keynote lecture. *Gen Thorac Cardiovasc Surg*. 2016;64(7):380–5. 27130186 10.1007/s11748-016-0650-3 PMC4916188

[22] Tanaka K, Yamasaki M, Sugimura K, et al. Thoracic duct resection has a favorable impact on prognosis by preventing hematogenous spread of esophageal cancer cells: a multi-institutional analysis of 2269 patients. *Ann Surg Oncol*. 2021;28(8):4402–10. 33861403 10.1245/s10434-021-09962-4

[23] van der Horst S, Weijs TJ, Ruurda JP, et al. Robot-assisted minimally invasive thoraco-laparoscopic esophagectomy for esophageal cancer in the upper mediastinum. *J Thorac Dis*. 2017;9(Suppl 8):S834–42. 28815081 10.21037/jtd.2017.03.151 PMC5538971

[24] van der Sluis PC, Ruurda JP, Verhage RJ, et al. Oncologic long-term results of robot-assisted minimally invasive thoraco-laparoscopic esophagectomy with two-field lymphadenectomy for esophageal cancer. *Ann Surg Oncol*. 2015;22(Suppl 3):S1350-1356. 26023036 10.1245/s10434-015-4544-x PMC4686562

[25] de Groot EM, van der Horst S, Kingma BF, et al. Robot-assisted minimally invasive thoracolaparoscopic esophagectomy versus open esophagectomy: long-term follow-up of a randomized clinical trial. *Dis Esophagus*. 2020;33(Suppl):2. 10.1093/dote/doaa079 33241302

[26] Wada H, Doki Y, Nishioka K, et al. Clinical outcome of esophageal cancer patients with history of gastrectomy. *J Surg Oncol*. 2005;89(2):67–74. 15660375 10.1002/jso.20194

[27] Watanabe M, Mine S, Nishida K, Kurogochi T, Okamura A, Imamura Y. Reconstruction after esophagectomy for esophageal cancer patients with a history of gastrectomy. *Gen Thorac Cardiovasc Surg*. 2016;64(8):457–63. 27234222 10.1007/s11748-016-0661-0

[28] Tanaka K, Yamasaki M, Yamashita K, et al. Safety and efficacy of double-tract reconstruction with remnant stomach conservation in esophagectomy for patients with a history of distal gastrectomy. *Esophagus*. 2023;20(1):72–80. 36209181 10.1007/s10388-022-00958-x

[29] Dindo D, Demartines N, Clavien PA. Classification of surgical complications: a new proposal with evaluation in a cohort of 6336 patients and results of a survey. *Ann Surg*. 2004;240(2):205–13. 15273542 10.1097/01.sla.0000133083.54934.ae PMC1360123

[30] Tsuji T, Matsuda S, Sato Y, et al. Safety and efficacy of conversion therapy after systemic chemotherapy in advanced esophageal cancer with distant metastases: a multicenter retrospective observational study. *Ann Surg Oncol*. 2025;32(1):274–83. 39266786 10.1245/s10434-024-16196-7

[31] Azad TD, Chaudhuri AA, Fang P, et al. Circulating tumor DNA analysis for detection of minimal residual disease after chemoradiotherapy for localized esophageal cancer. *Gastroenterology*. 2020;158(3):494–505. 31711920 10.1053/j.gastro.2019.10.039 PMC7010551

[32] Zhang Q, Luo J, Wu S, et al. Prognostic and predictive impact of circulating tumor DNA in patients with advanced cancers treated with immune checkpoint blockade. *Cancer Discov*. 2020;10(12):1842–53. 32816849 10.1158/2159-8290.CD-20-0047 PMC8358981

[33] Iwaya T, Endo F, Takahashi F, Tokino T, Sasaki Y, Nishizuka SS. Frequent tumor burden monitoring of esophageal squamous cell carcinoma with circulating tumor DNA using individually designed digital polymerase chain reaction. *Gastroenterology*. 2021;160(1):463-465.e4. 33011175 10.1053/j.gastro.2020.09.035

[34] Morimoto Y, Matsuda S, Kawakubo H, et al. Tumor burden monitoring with circulating tumor DNA during treatment in patients with esophageal squamous cell carcinoma. *Ann Surg Oncol*. 2023;30(6):3747–56. 36788188 10.1245/s10434-023-13194-z

[35] Sun JM, Shen L, Shah MA, et al. Pembrolizumab plus chemotherapy versus chemotherapy alone for first-line treatment of advanced oesophageal cancer (KEYNOTE-590): a randomised, placebo-controlled, phase 3 study. *Lancet*. 2021;398(10302):759–71. 34454674 10.1016/S0140-6736(21)01234-4

