# Supplementary figures and images for: Outcome of Conversion Surgery After Induction Therapy for Esophageal Cancer with Synchronous Para-Aortic Lymph Node Metastasis: A Multi-institutional Retrospective Study
Source: Ann Surg Oncol. 2025 Aug 20;32(12):9270–80. doi: 10.1245/s10434-025-18042-w (PMC12534259; doi:10.1245/s10434-025-18042-w)

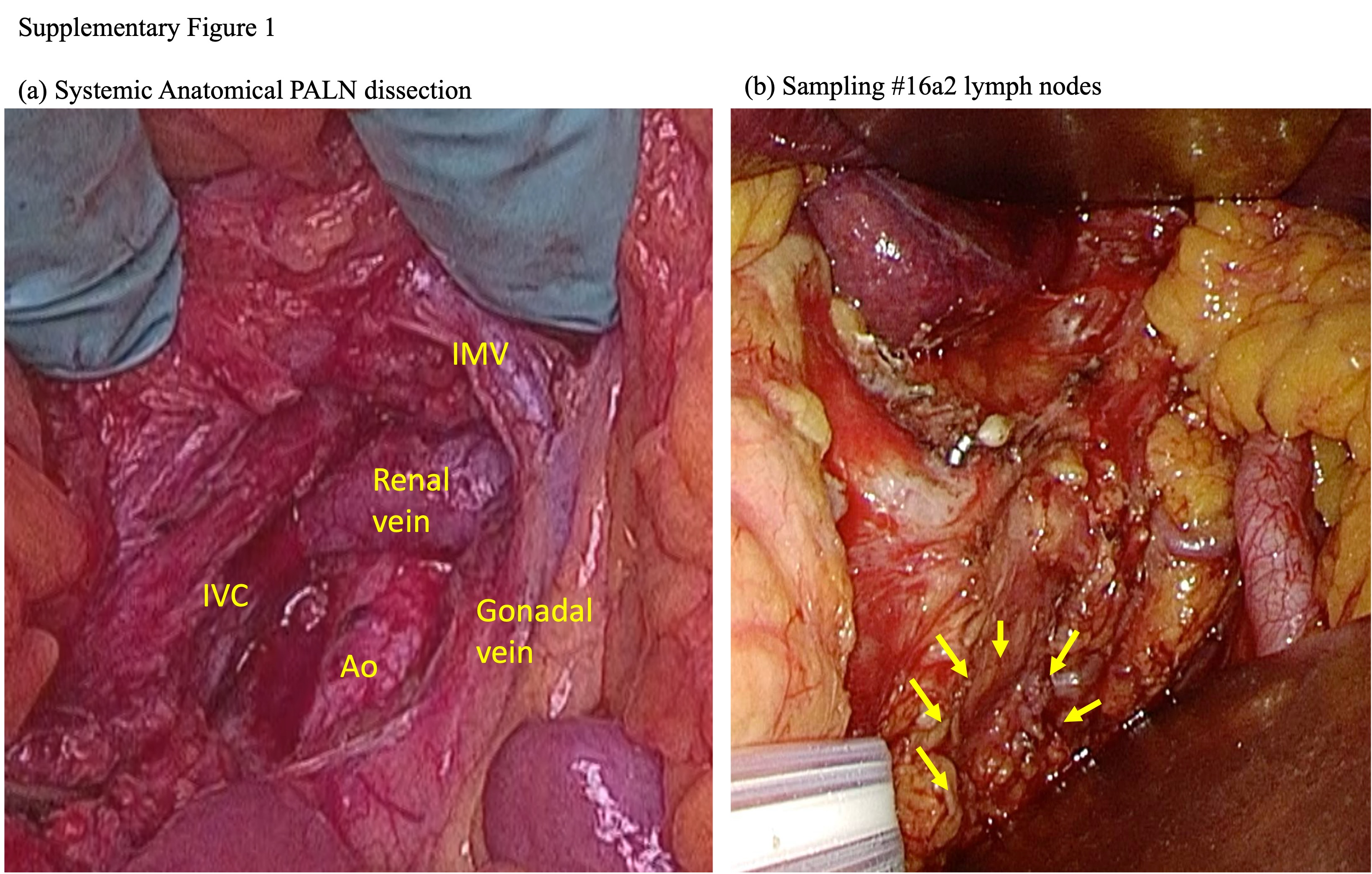

Supplement: Supplementary file 1 — Supplementary file1 (JPG 988 kb) [file 10434_2025_18042_MOESM1_ESM.jpg]
